# Asymmetries During Multiple Face Encoding: Increased Dwell Time and Number of Fixations in the Upper Visual Hemifield

**DOI:** 10.1177/2041669519827974

**Published:** 2019-02-13

**Authors:** Fatima M. Felisberti, Liam Currie

**Affiliations:** School of Behaviour and Social Sciences, Kingston University, London, UK

**Keywords:** eye movements, dwell time, visual asymmetry, visual field, face recognition

## Abstract

Visual field asymmetries in the encoding of groups of faces have rarely been investigated. Here, eye movements (percentage of dwell time [pDT] and number of fixations [nFix]) were recorded during the encoding of three groups of four faces tagged with cheating, cooperative, or neutral behaviours. Faces in each group were placed in the top left, top right, bottom left, or bottom right quadrants. Face recall was equally high in the three behavioural groups. Conversely, pDT and nFix were higher for faces in the upper hemifields. Most of the first saccades were made to the top left visual quadrant, which also commanded a higher pDT and nFix than the other quadrants. The findings are relevant to the understanding of visual field asymmetries in the processing of multiple faces, a common social scenario, and may be linked to reading habits in conjunction (or not) with cultural and environmental cues.

## Introduction

Face-to-face communication is at the core of human social life and it relies strongly on our ability to recognize the faces of people we have interacted with, not only to develop stable future associations and exchanges (Rand, Dreber, Ellingsen, Fudenberg, & Nowak, 2009) but also to avoid conflict or financial losses (Blais, Jack, Scheepers, Fiset, & Caldara, 2008; Bonner, Burton, & Bruce, 2003). Hence, a well-developed ability to recognize faces linked to distinct types of behaviour is evolutionarily advantageous.

Few studies examined face recognition with multiple faces at once and even fewer investigated tagging faces with different behaviours, a common behaviour in social gatherings and relevant information in eye witnesses’ reports. The findings from earlier studies showed a range of face recognition biases in social scenarios, from biases towards cooperators (cf., Barclay, 2004) or free-riders (cf., Cosmides, Tooby, Fiddick, & Bryant, 2005), to reports of no biases towards either of them (cf., Felisberti & Farrelly, 2016). One such study revealed recognition biases towards faces tagged with a cooperative rather than neutral or cheating behaviour (Felisberti & Pavey, 2010). Other studies suggest that faces tagged with less frequent behaviours are the faces that will be better recalled, independently of the type of behaviour associated with them. Yet, the question of whether we have any a priori bias is still open.

Recognition biases have also been associated with the location of faces in the visual field (Carlei & Kerzel, 2015). Furthermore, using a visual search paradigm to investigate gaze processing, Carlei, Framorando, Burra, and Kerzel (2017) reported upper and left visual field asymmetries (VFAs) that could be enhanced or suppressed by varying specific characteristics of the stimuli presented. This is not surprising since there are important and well-established variations in receptive field size across the retina, cortical magnification factors, and visual acuity, to cite just a few variables (Silva et al., 2018).

An upper visual hemifield advantage in face processing and a lower hemifield advantage in perceptual motion accuracy have been consistently reported in past studies using assorted experimental paradigms (Carlson, Hogendoorn, Fonteijn, & Verstraten, 2011; Christman, 1993; Hillger & Koenig, 1991; Luh, Rueckert, & Levy, 1991; Sergent, 1984; Stone & Valentine, 2005; Zito, Cazzoli, Müri, Mosimann, & Nef, 2016). Such VFAs (or biases) favoured the recall of faces presented in the upper and, to a lesser extent, the left hemifields (Felisberti & McDermott, 2013).

The origin of VFAs in face recall is not known. VFAs related to face processing may be modulated by environmental cues (e.g., illumination), cultural cues (e.g., language, reading) or cognitive cues (e.g., attention). The advantage of the top hemifield might be linked to prior visual knowledge of the environment and one’s peri-personal space, since most natural and artificial light comes from above our heads. Such knowledge could be used to disambiguate scenes and lead to processing biases towards upper, left-lit stimuli (Gerardin, de Montalembert, & Mamassian, 2007). Visuospatial attention may be at least partially responsible for such upper advantage, as shown by Quek and Finkbeiner (2016) using a masked face processing paradigm.

Despite the wide range of studies on eye movements and memory for faces, to date no study has investigated the eye movements of neurotypical adults during multiple face encoding, a frequent situation in social scenarios. Hence, this study examined if there were perceptual VFAs in eye movements during the encoding of multiple faces, as suggested by the different recognition accuracy for faces presented at different locations in the visual field (Felisberti & McDermott, 2013). Here, the number of fixations (nFix), the direction of the first saccade and the percentage of dwell time (pDT) were recorded for faces presented in four visual quadrants (top left [TL], top right [TR], bottom left [BL] and bottom right [BR]). Note that the terms top or upper and bottom or lower are used interchangeably in the literature. Eye movements were also monitored to examine eventual differences linked to the social behaviours tagged to those faces. The underlying assumption was that reading habits could be modulating VFAs in face processing, an assumption based on studies that attributed left hemifield biases to a right-hemisphere dominance for selective attention (Holcombe & Nguyen, 2017; Ullman, 1984).

## Method

### Participants

There were 40 participants (8 men and 33 women) with age ranging from 19 to 38 years (*M* = 22.76, standard deviation [*SD*] = 4.32). From the initial 42 participants, one was excluded due to problems during the calibration of the eye tracker and another participant was excluded from the nFix analysis due to outlier values. About two-third participants were university students and one third were members of the public living or working near the university premises. They were recruited via opportunity sampling and on a voluntary basis.

All participants provided written consent prior to testing in accordance with the ethical guidelines of the British Psychological Society and following the approval by the university ethics committee. Participants were informed that the experiment was about face recognition and that their eye movements would be recorded, but no further experimental details were given. Participation was not compensated financially, but some students received bonus course credits. All participants had normal or correct-to-normal vision.

## Materials

### Stimuli

An equal number of male and female facial photos was selected from the XM2VTS database (*N* = 24). The 12 faces to be memorized were divided in three groups tagged as cheaters, cooperators or neutrals (depending on their moral behaviour in a hypothetical financial transaction). The face recognition test was written using EyeLink’s integrated software. The faces on each of the behavioural groups were randomly allocated to one of four visual quadrants around a centred fixation point: TL, TR, BL and BR. The remaining 12 faces were used in the subsequent recall test. A pilot study showed the faces used here did not differ significantly from each other in a face recognition test (Felisberti & Aidoo, 2009). A chin rest was used to stabilize the participants’ head position at approximately 60 cm from the screen centre in a dimly lit room. The stimuli were presented on a 21″ LCD monitor with a resolution of 1280 × 1024 and 75 Hz as vertical refresh rate. The faces were inside areas of interest on each of the four visual quadrants with a viewing angle of approximately 5° × 7° (width vs. height).

### Eye movements

The observer’s left or right eye movements were recorded using a video-based eye tracker with a spatial resolution of 0.1° (Eyelink 1000; SR Research, Ontario, Canada). For the scan path analysis, regions were defined using Data Viewer software supplied by SR Research. The eye tracker was calibrated with the 12-point procedure. Eye movements were sampled at a rate of 1000 Hz and eye position was sampled automatically (500 times/second). Eye gaze data were analysed in two stages. Saccades were identified using the default settings of EyeLink 1000’s automatic parser. An eye movement was classified as saccade if it had an instantaneous velocity of greater than 30°/second, or an acceleration greater than 8,000°/second^2^, with all remaining data points between successive saccades were classified as fixations. Dwell time is defined as the total time spent viewing (fixating) each face during a trial and expressed as percentages. The EyeLink Data Viewer software was then used to calculate the mean pDT, the first saccade to an area of interest and the mean nFix to each area of interest.

### Procedure

After being briefed about the study and signing a consent form, participants were tested in a quiet room and sat in a height-adjustable chair to prevent any rotation about the vertical axis. Participants were tested individually and according to the following core experimental protocol: (a) encoding of 12 faces with correspondent behaviours, (b) distracter task during memory consolidation (10 multiplications) and (c) ‘yes-no’ face recognition test (24 trials: 12 encoded and 12 new ones).

The 12 faces in the encoding phase were divided in three behavioural groups with four faces each: cheaters, cooperators and neutrals. The social scenario was based on a fictitious character (‘John’) able to lend his friends £2,500,000. Some friends borrowed the money and paid it back with interest after a year (cooperators), some borrowed it but never paid it back (cheaters) and some never borrowed any money from John (neutrals). The faces were counterbalanced and randomly presented in the different quadrants and groups across participants.

The simple recall phase started with a face at the centre of a screen and the question ‘Have you seen this face before?’ Half of the faces had been memorized in the encoding phase, and half of the faces were new (i.e., absent from the encoding phase). Participants answered by pressing a key (1 = *Yes*; 2 = *No*). A cycle of 24 faces (12 memorized and 12 new) was presented in randomized order to each participant. The whole procedure lasted 10 to 15 minutes. For further details, see Felisberti and McDermott (2013).

### Data Analysis

In the absence of previous studies on VFAs using a similar experimental paradigm, the choice of sample size in this study was loosely based on the number of participants recruited in related eye movement and contextual face recognition studies.

The datasets were assessed for normality and means were accepted as having a normal distribution if the kurtosis fell in the range of ± 2.0. The analysis of variance (ANOVA) had the descriptors tagged to faces (cooperators, cheaters and neutrals) and the visual hemifields (top vs. bottom and left vs. right) as the independent variables and the pDT and nFix as dependent variables. Greenhouse–Geisser adjustments to the degrees of freedom were performed when sphericity could not be assumed (Mauchly’s sphericity test). Bonferroni adjustments were used in all pairwise comparisons. The partial eta-squared (pη^2^) was used to refer to effect size (Levine & Hullett, 2002). The following ‘rules of thumb’ were used to evaluate effect size: .01 small or modest, .06 medium or moderate and .14 large, but such cut-off values should not be taken as precise boundaries (Cohen, 1990, 1992).

## Results

### Cheaters Versus Cooperators

#### Recall accuracy

An easier and shorter version of the face recall test used in an earlier study (Felisberti & McDermott, 2013) was used here to ‘back-monitor’ if participants (*N* = 41) had attended to the faces during the encoding phase. The accuracy to faces tagged with three behavioural conditions was high and statistically similar, *F*(2, 80) = .38, *p* = .684, pɳ^2 ^= .01. The mean accuracy and *SD* were as follows: *Cheaters* 85% ± 2, *Cooperators* 86% ± 2 and *Neutrals* 89% ± 2. The relatively high accuracy levels suggest that attentional resources were deployed efficiently during the preceding face encoding phase.

A response sensitivity analysis (*d* prime or *d′*) confirmed that the mean perceptual sensitivity to the tagged faces was indeed similar in the three conditions, *F*(2, 80) = .37, *p* = .69, pɳ^2 ^= .01 (*Cheaters*, *d*′ = 2.51 ± 1.19; *Cooperators, d*′ = 2.55 ± 1.09; and *Neutrals, d*′ = 2.68 ± 1.11).

#### Eye movements with tagged faces

The overall time spent by the participants looking at the faces of cheaters, cooperators and neutrals was statistically similar, *F*(2,80) = .31, *p* = .73, pɳ^2 ^= .01, which is in line with the recall accuracy for those faces. The mean pDT ( ± *SD*) for each behavioural condition were *Cheaters* (23% ± 5), *Cooperators* (23% ± 3) and *Neutrals* (23% ± 5).

A 4 (quadrants) × 3 (tagged behaviours) ANOVA showed no significant interaction between dwell time and the behaviours tagged to the faces in each quadrant, *F*(6,240) = 1.04, *p* = .40, pɳ^2 ^< .03.

The presence of left or right visual hemifield asymmetries was tested with a 2 (left × right hemifield) × 3 (tagged behaviours) ANOVA. Although pDT in the left hemifield tended to be higher than in the right hemifield, the difference was not significant across behaviours, *F*(2,80) = .66, *p* = .52, pɳ^2 ^< .02. A similar 2 (top × bottom hemifield) × 3 (tagged behaviours) ANOVA revealed no significant interactions between pDT and behaviours across the top and bottom hemifields, *F*(2,80) = .62, *p* = . 54, pɳ^2 ^= .02.

### Eye Movements During Face Encoding

Most of the first saccades were made to the TL quadrant (78%). Only a few of the first saccades were directed to the TR (10%), the BL (8%) or the BR (2%) quadrants or to the fixation point (2%) at the centre of the screen.

Since there were no reliable differences in eye movements to the faces tagged with cheating, cooperative or neutral behaviours, those datasets were merged according to the visual quadrants in which the faces were displayed. The findings with the aggregated dataset are presented later.

#### Visual quadrants

##### pDT

**Figure 1.**
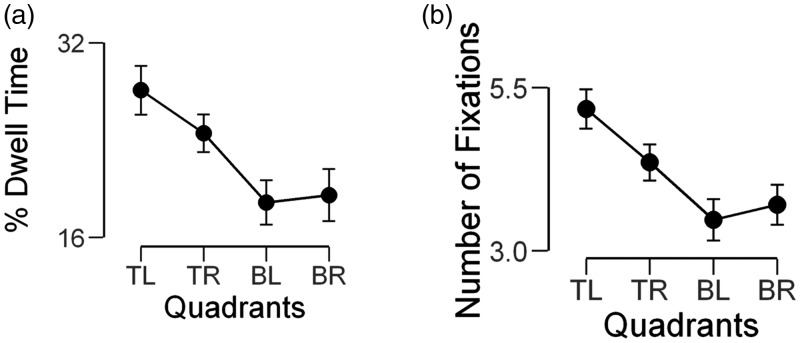
Visual field asymmetries in eye movements during multiple face encoding. The percentage of dwell time (a) and the number of fixations (b) in four visual quadrants: TL, TR, BL and BR. The bars show the 95% confidence intervals. TL = top left; TR = top right; BL = bottom left; BR = bottom right.

The averaged pDT values for each quadrant are given in Table 1. There was a significant main effect of time spent looking at each quadrant, *F*(3,117) = 21.95, *p* < .001, pɳ^2 ^= .36. Pairwise comparisons revealed that participants looked longer at faces in the TL quadrant than in the TR (*p* = .03), BL (*p* < .001) or BR (*p* < .001) quadrants. The pDT in the BL and BR quadrants was similar (*ps* = 1) (Figure 1(a)).

**Table 1.** The Mean pDT and nFix During the Encoding of Faces Located in the Top Left, Top Right, Bottom Left or Bottom Right Visual Quadrants As Well As the Aggregated Mean Values for the Left and Right and Top and Bottom Hemifields (Mean ± *SE* [95% Confidence Interval]).

	pDT		nFix	
Top left	28% ± 1%	[26, 30]	5.18 ± 0.14	[4.89, 5.46]
Top right	25% ± 1%	[23, 26]	4.36 ± 0.15	[4.05, 4.66]
Bottom left	19% ± 1%	[17, 21]	3.48 ± 0.16	[3.16, 3.79]
Bottom right	19% ± 1%	[17, 22]	3.71 ± 0.18	[3.35, 4.07]
Top hemifield	26% ± 1%	[25, 27]	4.77 ± 0.12	[4.53, 5.00]
Bottom hemifield	19% ± 1%	[18, 21]	3.59 ± 0.13	[3.32, 3.86]
Left hemifield	24% ± 1%	[22, 25]	4.33 ± 0.10	[4.13, 4.53]
Right hemifield	22% ± 1%	[21, 23]	4.03± 0.13	[3.77, 4.29]

*Note.* pDT = percentage of dwell time; nFix = number of fixations.

##### nFix

The mean nFix varied with the quadrant in which the faces were encoded, *F*(3,117) = 26.29, *p* < .001, pɳ^2 ^= .40. The nFix for faces in the TL quadrant was higher than in the TR, BL and BR quadrants (*p* < .001). As observed with pDT, the nFix in the BL and BR quadrants was also statistically similar (*ps* = 1) (Table 1, Figure 1(b)).

#### Visual hemifields: Left versus right

##### pDT

There was no difference in the mean pDT between the right and left hemifields, *F*(1,39) = 1.88, *p* = .18, pɳ^2 ^= .05, even though the pDT in the left hemifield was slightly higher than in the right hemifield.

##### nFix

The mean nFix in the left was significantly higher than in the right hemifields, *F*(1,39) = 4.34, *p* < .04, pɳ^2 ^= .10 (Table 1).

#### Visual hemifields: Top versus bottom

##### pDT

Contrary to the observed with the left and right hemifields, a marked difference was observed between the mean pDT for the top and bottom hemifields, *F*(1,39) = 57.43, *p* < .001, pɳ^2 ^= .60 (Table 1), with the top value higher than the bottom one.

##### nFix

A similar significant difference was also observed between the mean nFix in the top and lower hemifields, *F*(1,39) = 46.95, *p* < .001, pɳ^2 ^= .55 (Table 1).

## Discussion

VFAs in face processing have been reported in studies using a wide range of experimental paradigms, and many showed an advantage of the upper visual field for object recognition and faces. Nonetheless, to our knowledge, this is the first study to address unanswered questions from previous studies by examining if VFAs in eye movements during the encoding of groups rather than individual faces varied with their location in different visual quadrants and hemifields.

The first set of findings showed that social behaviours tagged to faces did not affect the time participants spent looking at them during the encoding phase. The same was true in the recall test, with similar accuracy for faces tagged as belonging to cheaters, cooperators or neutrals. Those findings contradict an earlier study showing a memory advantage for the faces of *cooperators* over *cheaters* (Felisberti & McDermott, 2013), which is not entirely surprising since the recall test in the previous study was more difficult, with 72 trials rather than the 24 trials in this study. In this easier test, the mean accuracy may have been too close to ceiling values (85%–89%) to reveal any reliable effect of the behavioural tags, and a study with more trials in the recall phase is needed to check if the face recall was indeed similar.

Since there were no significant differences in pDT and nFix associated to behavioural tags, the second set of findings focused on the aggregated eye movements to faces encoded in different visual quadrants and hemifields. The findings revealed a clear advantage of the TL quadrant during face encoding, both in terms of pDT and nFix, which is particularly relevant to cases where several faces need to be processed for short periods of time (e.g., surveillance, visual search, web design, etc.).

The upper left quadrant pDT and nFix advantage during face encoding is supported by neurophysiological studies. For example, Zito et al. (2016) suggested that the dorsal visual stream processing motion perception has a bias for the lower visual hemifield, while the ventral visual stream processing shape perception has a bias for the upper visual hemifield. Furthermore, left hemifield superiority for faces has been linked to the activation of the right face fusiform region (Hines, Jordan-Brown, & Juzwin, 1987; Kanwisher, McDermot, & Chun, 1997; Thomas & Elias, 2011; Yovela, Tambini, & Brandman, 2008). Right-hemisphere dominance has also been linked to left hemifield holistic face processing and gaze processing (Hillger & Koenig, 1991; Rossion et al., 2000).

Strong evidence supporting the current findings comes also from an assorted array of behavioural studies. Felisberti and McDermott (2013) examined VFAs by measuring the recognition accuracy to faces encoded in different visual quadrants and reported higher accuracy for faces encoded in the top quadrants. An upper hemifield advantage was also reported in face matching tasks, albeit on the right rather than left visual hemifield (Hagenbeek & Van Strien, 2002), which might be linked to the feature-based processing associated with left-hemisphere dominance (Gauthier, Curran, Curby, & Collins, 2003).

VFAs have been associated with a multitude of different factors, from visuospatial attention and brain lateralization of face processing, to reading habits (Rayner, 2009) and prior knowledge of the environment. In addition, Previc (1990) used the point of gaze as a core reference and proposed that images presented above it were analysed ‘offline’ and linked to perceptual categorization, while images below the point of gaze were subjected to an ‘online’ analysis and associated with motor control. VFAs in favour of the top hemifield might also be linked to prior visual knowledge of the environment and one’s peri-personal space, since most natural and artificial light comes from above our heads. Such knowledge could be used to disambiguate scenes and lead to processing biases towards top-lit stimuli (Gerardin et al., 2007; Mamassian & Goutcher, 2001).

The role played by the reading of the behavioural tags required before the encoding of each group of faces has not been directly addressed in this study, and a series of studies are under way to investigate the role of language in VFAs in eye movements. Nonetheless, many studies showed an advantage of the upper hemifield in the recognition of letters and words (Foulsham, Gray, Nasiopoulos, & Kingstone, 2013; Goldstein & Babkoff, 2001; Hagenbeek & Van Strien, 2002; Holcombe & Nguyen, 2017). Taking into account such findings and the fact that in this study about three fourth of the first saccades were directed to the TL quadrant, and nFix and pDT were higher in the upper hemifields, it is possible that reading habits played an important modulatory role in the VFAs reported here, either alone or in conjunction to other environmental cues, since they are believed to be linked to flexible attentional prioritization.

## Informed Consent

Informed consent to execute genetic analysis and to publish Videos have been obtained by the patient’s guardian.

## Supplementary Material

Supplementary material

Supplementary material
