# Corrigendum

**DOI:** 10.1177/2041669519853646

**Published:** 2019-05-20

Felisberti, F. M., & Currie, L. (2019). Asymmetries during multiple face encoding: Increased dwell time and number of fixations in the upper visual hemifield. *i-Perception*, *10*(1), 1–10. doi:10.1177/2041669519827974

The authors regret that they incorrectly recorded the following article in the References on page 9:

Holcombe, A. O., & Nguyen, E. H. L. (2017). Implied reading direction and prioritization of letter encoding. *Journal of Experimental Psychology: General*, 146, 1420–1437.

The correct reference is as follows:

Holcombe, A. O., Nguyen, E. H. L. & Goodbourn, P. T. (2017). Implied reading direction and prioritization of letter encoding*. Journal of Experimental Psychology: General*, *146*, 1420–1437.

This relates to the citations within the text on page 2 and 7 which should read as follows:

Holcombe, Nguyen & Goodbourn, 2017

